# An Interesting Case of Methicillin-Resistant Staphylococcus aureus Prostate Abscess in an Immunocompetent Patient

**DOI:** 10.7759/cureus.43131

**Published:** 2023-08-08

**Authors:** Nida Ansari, Sacide S Ozgur, Rhea Bhargava, Ryan Rahman, Bing Gong

**Affiliations:** 1 Internal Medicine, St. Joseph's Regional Medical Center, Paterson, USA

**Keywords:** staph aureus, mrsa, bacteremia, prostate abscess, prostatitis

## Abstract

Prostate abscess is a rare complication of prostatitis, typically observed in patients with conditions such as immunodeficiency, diabetes, urinary tract abnormalities, and chronic indwelling catheters. Gram-negative bacteria such as Enterobacteriaceae are the most commonly detected organisms in prostate abscesses. Methicillin-resistant *Staphylococcus aureus* (MRSA) infections are rarely reported. The unique aspect of our case involves MRSA bacteria, further complicated by an MRSA prostate abscess, in a 61-year-old immunocompetent male. The patient, with a past medical history of hypertension and diabetes, presented to the emergency department complaining of nausea and vomiting for four days, with an associated subjective fever and right-sided abdominal pain. A computed tomography (CT) scan of the abdomen/pelvis with contrast showed a prostatic abscess, with abscess/phlegmon extending bilaterally into the seminal vesicles. Urine and blood cultures were positive for MRSA. Initially, Piperacillin/Tazobactam and Vancomycin were initiated. Subsequently, the treatment was switched to Daptomycin. The patient also underwent cystoscopy with urethral dilation, transurethral prostate resection, and unroofing. Although MRSA is not a typical causative agent of prostatitis, it should be considered in the differential diagnosis, especially when clinical improvement cannot be achieved with standard empirical treatment. Timely identification and appropriate treatment (such as drainage and antibiotics) are crucial for both patient survival and the prevention of complications.

## Introduction

Prostatitis is an inflammation of the prostate, which can be categorized into four subtypes: acute bacterial prostatitis, chronic bacterial prostatitis, chronic prostatitis/chronic pelvic pain syndrome (CP/CPPS), and asymptomatic inflammatory prostatitis [1]. Acute and chronic bacterial prostatitis are typically caused by gram-negative organisms, such as Enterobacteriaceae, and are often associated with urinary tract infections or sexually transmitted infections. Symptoms usually include dysuria, lower back pain, and increased urinary frequency. These conditions can lead to complications, including prostate abscesses (PAs), bacteremia, and pyelonephritis if left untreated [1]. CP/CPPS, the most common form of the disease, can present with a wide array of symptoms and might occur without an identifiable bacterial infection. On the other hand, asymptomatic inflammatory prostatitis is characterized by inflammation without any genitourinary symptoms [2]. Prostatitis is the leading cause of urinary tract problems in men under 50 years of age and the third most common urinary tract issue for those over 50 [1]. While the most common cause of prostatitis/PAs is gram-negative bacteria, methicillin-resistant *Staphylococcus aureus* (MRSA), a gram-positive bacteria, has gained attention for its potential involvement in these cases and its resistance to many commonly used antibiotics such as methicillin and other beta-lactam antibiotics [3]. MRSA is a pathogen increasingly associated with a wide range of clinical syndromes. However, its involvement in causing prostatitis and PA is relatively rare and vastly underreported [3]. PA typically arises from an infection that begins in the prostate gland, which can occur due to bacterial invasion through the urethra, direct spread from nearby structures, or bloodstream dissemination from a distant site of infection. MRSA in urine, while uncommon, have been reported and are generally associated with urinary tract abnormalities, catheter-associated infections, or as part of a systemic MRSA infection. This case report presents a 61-year-old immunocompetent male with spontaneous MRSA prostatitis complicated by a PA and bacteremia.

## Case presentation

A 61-year-old Hispanic male with a past medical history of hypertension and diabetes presented to the emergency department complaining of four days of nausea and vomiting, with associated subjective fever and right-sided constant abdominal pain. He described the pain as sharp and rated the pain 10/10. On the initial day, he reported having seven episodes of non-bloody and non-bilious vomiting. He mentioned being unable to tolerate oral intake and not having had a bowel movement for two days. Additionally, he reported experiencing dysuria for the past two days. He denied having had contact with anyone who was ill, consuming new foods, or recent travel. There were no reports of hematuria, hematochezia, chest pain, or shortness of breath. He also denied using intravenous (IV) drugs. He was not on any regular medication, and his medical, surgical, family, sexual, and social history was unremarkable.

**Figure 1:**
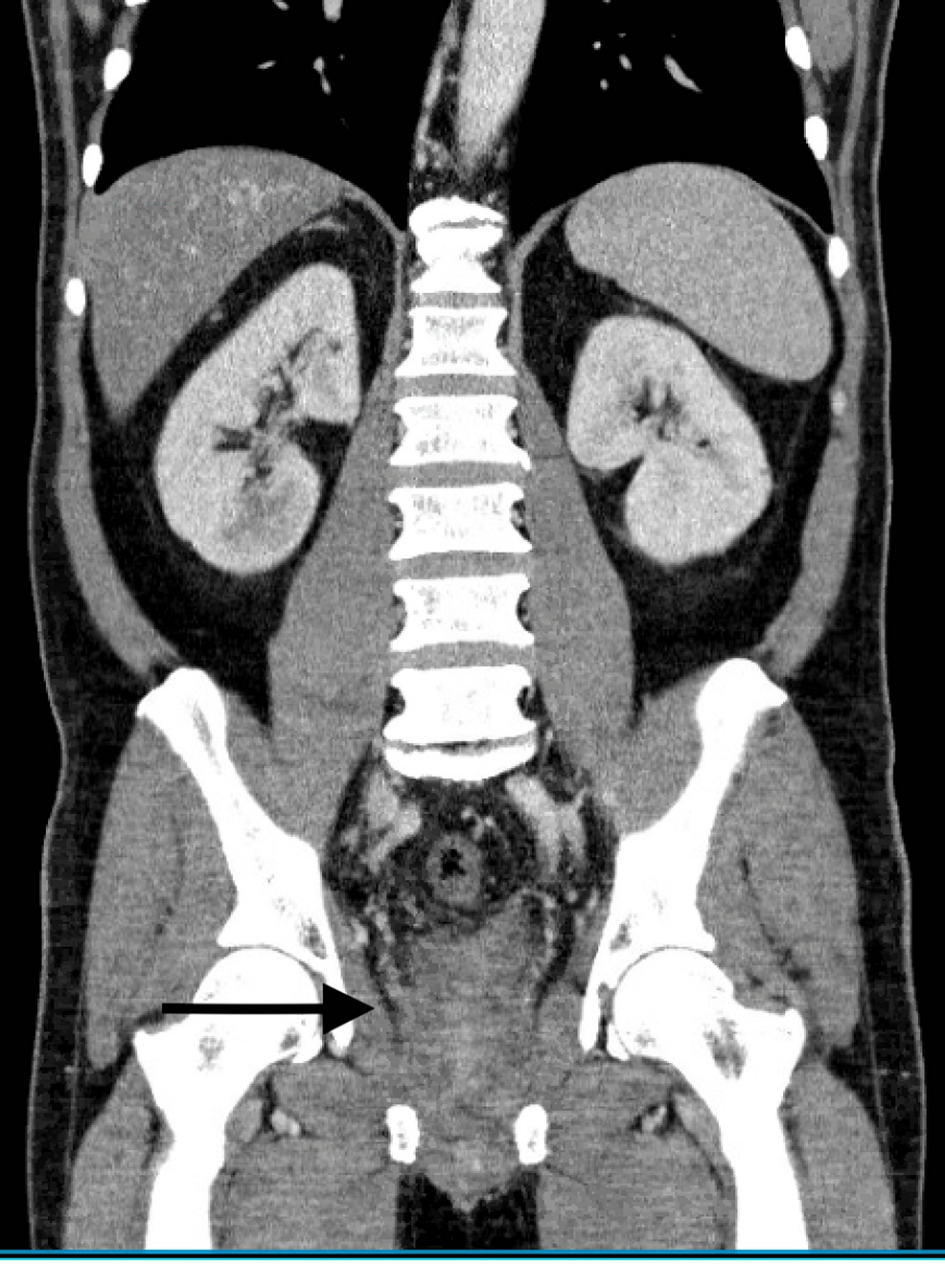
A significant thickening in the distal sigmoid and rectum with severe perirectal edema and thickening of the mesial rectal fascia. Prostate is moderately enlarged.

On physical examination, the patient was febrile at 38.5 𐩑C and tachycardic at 114 bpm, but his vitals were otherwise stable. The physical examination was remarkable for dry mucous membranes with a soft abdomen tender to palpation in the right upper quadrant and right lower quadrant. No rebound tenderness, guarding, or rigidity was noted. A digital rectal examination was not performed as the patient denied any symptoms of gastrointestinal (GI) bleeding. Initial workup revealed a complete blood count significant for leukocytosis with a left shift (WBCs 17.2 × 10^3 ^mm^-3^) and elevated hemoglobin A1c of 11.7%. The renal and hepatic functions were normal. Urinalysis showed a high specific gravity (>1.030) along with moderate ketones, a significant presence of blood, protein levels exceeding 500 mg/dL, and positive results for nitrites and leukocyte esterase. An initial CT scan was obtained that revealed an enlargement of the prostate and significant thickening of the distal sigmoid and rectum with severe perirectal edema, along with suspicious findings indicative of proctitis (Figure 1). 

The patient was admitted to the medical floor for treatment of proctitis and was started on piperacillin/tazobactam. Both urine and blood cultures on admission returned positive for MRSA. Vancomycin was added for bacteremia. He was also started on tamsulosin for benign prostate hyperplasia. He subsequently developed non-bloody diarrhea and rectal pain with defecation, which was indicative of proctitis with proctalgia, and a GI polymerase chain reaction (PCR) panel was obtained that was negative as well. His diarrhea resolved within a few days from admission with no intervention other than IV hydration to maintain euvolemia. A transthoracic and transesophageal echocardiogram was performed without significant vegetation noted or other evidence of endocarditis. Repeat blood cultures five days later were positive for MRSA. Vancomycin and piperacillin/tazobactam were switched to daptomycin due to its improved penetration for MRSA, given the presence of persistent positive blood cultures.

**Figure 2:**
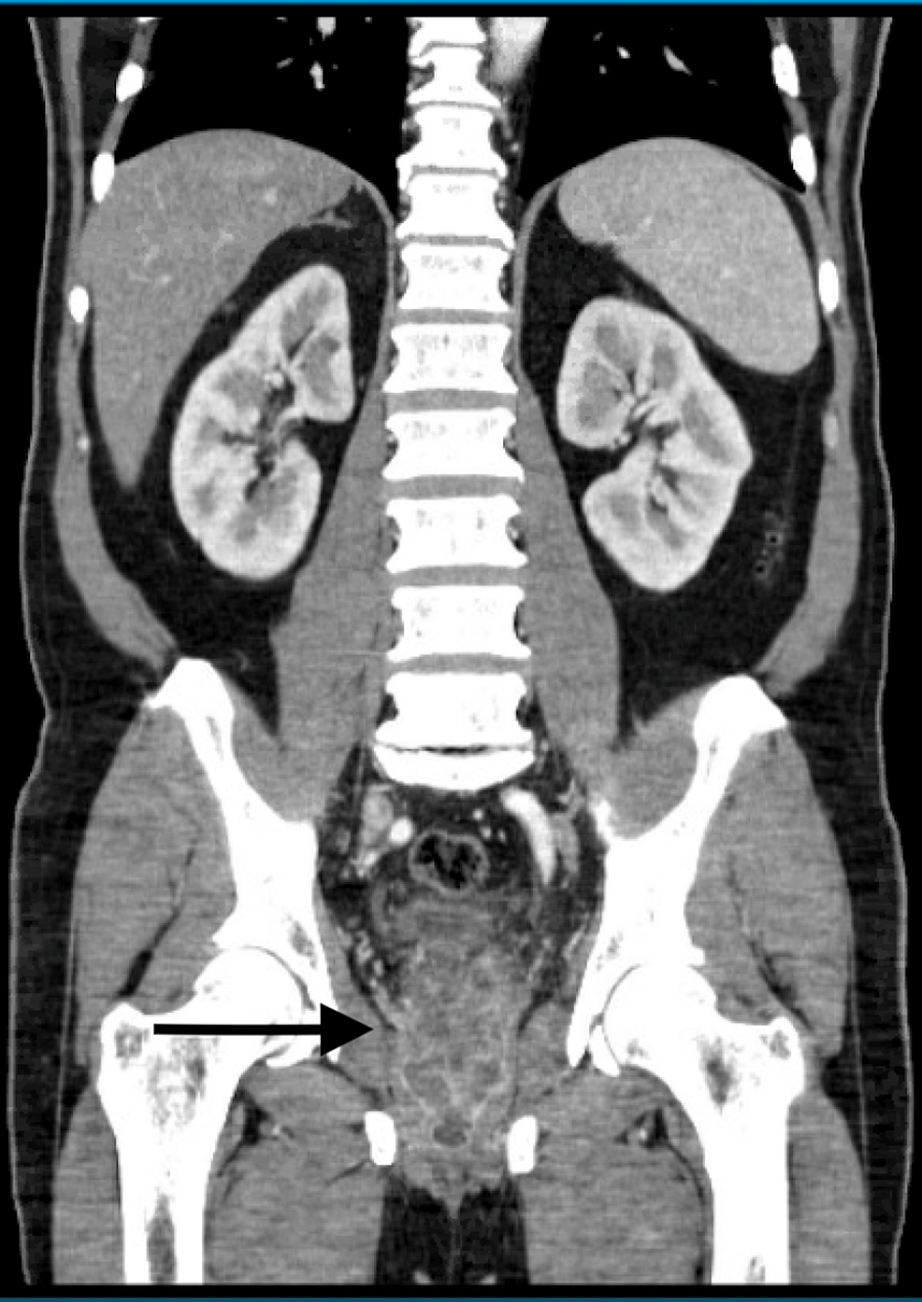
Rim-enhancing structure seen within the prostate bilaterally suspicious for prostatic abscesses measuring up to 2.0 cm × 1.2 cm on the left and 2.8 cm × 1.4 cm on the right, which appear to communicate in the midline.

A whole-body nuclear medicine bone imaging scan was obtained due to persistent bacteremia and was negative in determining an etiology. The patient’s WBC count continued to trend downward following the medication adjustment. Due to persistent positive blood cultures even two weeks after admission, repeat CT imaging was done that revealed a prostatic abscess/phlegmon extending into the bilateral seminal vesicles (Figure 2). Ceftaroline was subsequently added to the medication regimen for seven days for salvage therapy. The patient underwent cystoscopy with urethral dilation, transurethral prostate resection, and unroofing. Within a few days after the procedure, his clinical status improved. The patient was discharged with outpatient IV daptomycin infusions for two additional weeks due to the resolution of the infection and negative repeat blood cultures.

## Discussion

PAs are a rare entity, typically a complication of acute bacterial prostatitis that was not treated adequately or entirely. While more prevalent in men in their fifth or sixth decades, it can, however, affect individuals of any age [4]. Risk factors include dialysis patients, the presence of chronic indwelling catheters, immunocompromised patients, those undergoing urethral instrumentation, and diabetes mellitus, which recently has been identified as the most common predisposing risk factor for PA [4]. Gram-negative bacteria or Enterococcus species often cause PA [5]. A unique aspect of our case is the development of PA secondary to MRSA infection.

The presentation of PA is similar to acute bacterial prostatitis, which presents with acute onset of constitutional symptoms such as fever, chills, fatigue, low back/rectal/perineal pain, and urinary symptoms such as dysuria with frequency and urgency. A digital rectal exam (DRE) typically reveals a tender and enlarged gland. Fluctuance on DRE is often pathognomonic for PA [4]. Urinalysis typically reveals normal WBC counts with occasional instances of hematuria. Prostate-specific antigen (PSA) can be elevated as well [5].

Regarding the diagnosis of PA, it can be accomplished using transrectal ultrasound (TRUS), pelvic CT scan, or MRI [5]. TRUS successfully diagnoses PA in 80% to 100% of cases. However, due to patient discomfort, CT or MRI scans might be more optimal alternatives [4]. Clinicians should suspect possible MRSA infection in cases where there is no clinical improvement following antibiotic treatment with only Gram-negative coverage, as this is often the cause of PA [6]. Broadening the coverage to include MRSA is important, especially in individuals with a higher risk for MRSA PA.

While *S. aureus* infections are often implicated in many bone, heart, skin, and soft tissue infections, there has been a growing number of reports indicating its involvement in PA. While still exceedingly rare, it has been shown to be more prevalent in those with diabetes mellitus or immunocompromised patients [7]. Upon literature review, fewer than 50 cases reporting PA secondary to MRSA could be identified; however, this points toward a rising trend of MRSA as a cause of PA. The most common way of acquisition is hematogenous spread, and *S. aureus* is the most common bacteria to spread in this manner. Hematogenous spread is the predominant mode of acquisition, with *S. aureus* being the most frequent bacterium to disseminate in this manner. Generally, these patients exhibit *S. aureus* infection associated with conditions such as rheumatic fever, extensive furunculosis, chronic gingivitis, or osteomyelitis [4].

While there are no guidelines on PA secondary to MRSA infections, the general approach involves administering antibiotics and draining the abscess [3]. Resection may be required if the drainage is inadequate or if the abscess is larger than 1 cm [5]. Ensuring appropriate antibiotic coverage is vital. The prognosis is generally positive for these patients when promptly treated. Clinicians should include PA in their differential diagnosis, especially in individuals with risk factors such as diabetes mellitus, which is highly prevalent.

## Conclusions

This case report illustrates a rare but significant occurrence of MRSA prostatitis complicated by PA and bacteremia in an immunocompetent individual. It serves as a reminder that while MRSA is not a typical causative agent of prostatitis; it should be considered in the differential diagnosis when clinical improvement is not observed with standard empirical treatment. MRSA infections, owing to their resistance to many classes of antibiotics, pose significant treatment challenges, often leading to poorer clinical outcomes. Timely identification and appropriate treatment are crucial for patient survival and prevention of complications such as PA and bacteremia, especially in hospital settings or among individuals with risk factors such as underlying immunodeficiency, diabetes mellitus, and urinary tract abnormalities.
